# Correlation Analysis between Gut Microbiota and Metabolites in Children with Systemic Lupus Erythematosus

**DOI:** 10.1155/2021/5579608

**Published:** 2021-07-23

**Authors:** Min Wen, Taohua Liu, Mingyi Zhao, Xiqiang Dang, Shipin Feng, Xuewei Ding, Zhiquan Xu, Xiaoyan Huang, Qiuyu Lin, Wei Xiang, Xiaoyan Li, Xiaojie He, Qingnan He

**Affiliations:** ^1^Institute of Pediatrics, The Second Xiangya Hospital, Central South University, Changsha, China; ^2^Laboratory of Pediatric Nephrology, Institute of Pediatrics, The Second Xiangya Hospital, Central South University, Changsha, China; ^3^Department of Pediatrics, The Third Xiangya Hospital, Central South University, Changsha, Hunan, China; ^4^Department of Pediatric Nephrology, Chengdu Women's and Children's Central Hospital, School of Medicine, University of Electronic Science and Technology of China, Chengdu, Sichuan, China; ^5^Hainan Maternal and Children's Medical Center, Haikou, Hainan, China

## Abstract

Systemic lupus erythematosus (SLE) is an autoimmune-mediated diffuse connective tissue disease characterized by immune inflammation with an unclear aetiology and pathogenesis. This work profiled the intestinal flora and faecal metabolome of patients with SLE using 16S RNA sequencing and gas chromatography-mass spectrometry (GC-MS). We identified unchanged alpha diversity and partially altered beta diversity of the intestinal flora. Another important finding was the increase in Proteobacteria and Enterobacteriales and the decrease in Ruminococcaceae among SLE patients. For metabolites, amino acids and short-chain fatty acids were enriched when long-chain fatty acids were downregulated in SLE faecal samples. KEGG analysis showed the significance of the protein digestion and absorption pathway, and association analysis revealed the key role of 3-phenylpropanoic acid and *Sphingomonas*. *Sphingomonas* were reported to be less abundant in healthy periodontal sites of SLE patients than in those of HCs, indicating transmission of oral species to the gut. This study contributes to the understanding of the pathogenesis of SLE disease from the perspective of intestinal microorganisms, explains the pathogenesis of SLE, and serves as a basis for exploring potential treatments for the disease.

## 1. Introduction

Systemic lupus erythematosus (SLE) is an autoimmune-mediated diffuse connective tissue disease characterized by immune inflammation [1]. The presence of antinuclear antibodies in the serum and multisystem involvement are the two main clinical features of SLE, and the incidence rate of SLE in women is significantly higher than that in men [2]. Lupus nephritis (LN) occurs when SLE is complicated by renal damage. Approximately 60% of SLE patients suffer from LN complications that cause a higher mortality than SLE patients without LN [3]. At present, the aetiology and pathogenesis of SLE and LN are still unclear [4]. Studies have shown that the occurrence and development of some autoimmune diseases, such as inflammatory bowel disease [5, 6], rheumatoid arthritis [7, 8], and SLE [9, 10], are correlated with changes in intestinal microecology. Some studies found that the intestinal microbes of patients or animals with SLE were different from those of the control group through experimental models and clinical models [11–13], and the reconstruction of intestinal microecology through diet and drug regulation could help improve the severity of the disease [14]. There are many hypotheses on the interaction mechanism between intestinal microbes and the host and its relationship with SLE, but there are no mature theories yet. Currently, an increasing number of studies have shown that metabolites of the intestinal flora, such as short-chain fatty acids (SCFAs), free fatty acids (FFAs), amino acids, and arachidonic acid, correlate with autoimmune reactions [14–16]. However, the relationship between microbial metabolites and SLE remains unknown, and previously published studies are limited to adult patients. In this study, we collected the intestinal flora and metabolite data of 33 SLE children and 28 healthy controls (HCs) to clarify the pathogenesis of the disease from the perspective of intestinal microorganisms as much as possible, explain the pathogenesis of SLE, and serve as a basis for exploring potential treatments for the disease.

## 2. Material and Methods

### 2.1. Ethical Statement

All enrolled patients and healthy controls understood the sampling process and study plan in detail before sampling and signed informed consent forms. Ethical approval for this study was obtained from the Ethics Committee of the Second Xiangya Hospital of Central South University.

### 2.2. Study Subjects

A total of 28 healthy controls and 33 SLE patients were enrolled from the Second Xiangya Hospital of Central South University between December 2018 and April 2019. The diagnostic criteria of SLE were consistent with SLE classification criteria 2 revised by American College Rheumatology (ACR) in 1997 [17]. SLE patients were given hormone and/or immunosuppressive therapy. None of the selected subjects had applied or taken antibiotics, probiotics, symbiotic, or other microecological preparations for at least 3 months before sampling.

### 2.3. 16S rRNA Microbiota Analysis

Total genomic DNA from samples was extracted using the CTAB/SDS method. 16S/18S rRNA genes were amplified using primers including 16S V4-V5 : 515F-907R, 18S V9 : 1380F-1510R, and ITS1 : ITS1F-ITS2R. All PCRs were carried out in 30 *μ*l reactions with 15 *μ*l of Phusion® High-Fidelity PCR Master Mix (New England Biolabs), 0.2 *μ*M forward and reverse primers, and approximately 10 ng of template DNA. Thermal cycling consisted of initial denaturation at 98°C for 1 min, 30 cycles of denaturation at 98°C for 10 s, annealing at 50°C for 30 s, and elongation at 72°C for 60 s then 5 min. Electrophoresis was performed on a 2% agarose gel. PCR products were purified with GeneJET Gel Extraction Kit (Thermo Scientific). Sequencing libraries were generated using the NEBNext® Ultra™ DNA Library Prep Kit for Illumina (NEB, USA), and the library quality was assessed on the Qubit@ 2.0 Fluorometer (Thermo Scientific) and Agilent Bioanalyzer 2100 system. The library was sequenced on an Illumina MiSeq platform.

A total of 5507169 sequences were used for analysis. Sequence analysis was performed by the UPARSE software package using the UPARSE-OTU and UPARSE-OTUref algorithms. In-house Perl scripts were used to analyse the alpha diversity and beta diversity. Cluster analysis was preceded by principal component analysis (PCA). QIIME calculates both weighted and unweighted UniFrac distances. We used unweighted UniFrac distance for principal coordinate analysis (PCoA) and unweighted pair group method with arithmetic mean (UPGMA) clustering. Metastats software was utilized to confirm differences in the abundances of individual taxa between the two groups, and LEfSe was used for the quantitative analysis of biomarkers within different groups. To identify differences in microbial communities between the two groups, ANOSIM [18] and MRPP (multiresponse permutation procedure) [19] were performed based on Bray-Curtis dissimilarity distance matrices.

### 2.4. GC-MS Analysis

The instrumental status and balance as well as the stability of the gas chromatography-mass spectrometry system were evaluated using QC samples. Samples (60 mg) were mixed with 200 *μ*g water and vortexed for 60 s before being added to 800 *μ*l methanol acetonitrile (Merck, 1499230-935) solution (1 : 1, *v*/*v*), followed by vortexing for 60 s and low-temperature ultrasound for 30 min twice. Samples were stored at -20°C for 1 h to precipitate protein. Finally, the samples were centrifuged for 20 min at 14000 RCF at 4°C, and the supernatant liquor was collected for freeze drying and stored at -80°C.

For GC analysis, the samples were separated by an Agilent 1290 Infinity LC ultra-high-performance liquid chromatography (UHPLC) HILIC column at 25°C with a flow rate of 0.3 ml/min. The mobile phase was the composite of A (water, 25 mm ammonium acetate, 25 mm ammonium) and B (acetonitrile). Gradient elution procedures were as follows: B was 95% in 0-1 min, then changed linearly from 95% to 65% in 1-14 min and 65% to 40% in 14-16 min, and maintained at 40% in 16-18 min before changing linearly from 40% to 95% in 18-18.1 min, maintaining at 95% in 18.1-23 min. The samples were placed in an automatic sampler at 4°C throughout the analysis. A random sequence was used for the continuous analysis. QC samples were inserted into the sample queue for monitoring and evaluation of the system.

For Q-TOF MS analysis, electrospray ionized positive ions and negative ions were detected. The samples were separated by UHPLC and analysed by a Triple TOF 5600 mass spectrometer (AB SCIEX). The ESI source conditions after HILIC chromatographic separation were as follows: ion source gas 1 (Gas1): 60, ion source gas 2 (Gas2): 60, curtain gas (CUR): 30, source temperature: 600°C, ion spray voltage floating (ISVF) ±5500 V (positive and negative modes), TOF MS scan *m*/*z*range: 60-1000 Da, product ion scan *m*/*z*range: 25-1000 Da, TOF MS scan accumulation time 0.20 s/spectra, and product ion scan accumulation time 0.05 s/spectra. The second-order mass spectrum was obtained by information-dependent acquisition (IDA) with a high sensitivity mode, a declustering potential (DP): of ±60 V (positive and negative modes), collision energy of 35 ± 15 eV, and IDA excluded isotopes within 4 Da with 6 candidate ions to monitor per cycle.

The original data were converted into the mzXML format by ProteoWizard, and then, the XCMS program was used for peak alignment, retention time correction, and peak area extraction. Accurate mass number matching (<25 ppm) and secondary spectrogram matching were used to identify the metabolite structure, and the laboratory database was retrieved. After pretreatment by Pareto scaling, multidimensional statistical analysis was performed, including principal component analysis (PCA), supervised partial least square discriminant analysis (PLS-DA), and orthogonal partial least square discriminant analysis (OPLS-DA). One-dimensional statistical analysis included Student's *t*-test and multiple analysis of variation.

### 2.5. Association Analysis

The correlation analysis is mainly based on statistical algorithms to find the correlation between significantly different flora (*p* < 0.05) and the significantly different metabolites obtained through nontarget metabolomics analysis. Principal component analysis (PCA) and multivariate statistical analysis were performed with SIMCA version 14.1. KEGG pathway analysis was based on the online Kyoto Encyclopedia of Genes and Genomes (KEGG, http://www.kegg.jp/). Differentially abundant proteins/modified peptides/genes/and metabolites/lipids were log_2_ scaled (TMT/iTRAQ) or *Z*-score scaled (label free) and concatenated into one matrix. Then, the correlation coefficients among all the molecules in the matrix were calculated with the Pearson algorithm in R version 3.5.1. Cytoscape version 3.5.1 was used to calculate the correlation network.

## 3. Results

### 3.1. Clinical Features of SLE Patients

The basic characteristics of the participants at the time point of sample collection are listed in Table 1, including their sex, age, disease activity parameters, autoantibody status, and treatment. According to two-tailed unpaired Student's *t*-test, BMI was not significantly different between the SLE group and HC group (*p* = 0.234). All the children in the SLE group were also diagnosed with LN.

### 3.2. Intestinal Flora Is Different between SLE Patients and Healthy Groups

After data optimization, the average length of 99.87% of the sequence bars was within the range of 401-450 bp, and a total of 2,159 operational taxonomic units (OTUs) were retained for subsequent analysis.

Using a *t*-test, we compared the Chao1 index (*p* = 0.327) of the two groups and found no significant difference, suggesting that the species abundance does not change in SLE patients. Additionally, Shannon (*p* = 0.214), coverage (*p* = 0.988), and Simpson (*p* = 0.414) indexes that estimate community diversity also showed similar results. Taken together, these results show that the richness of the intestinal flora did not change among SLE patients.

**Figure 1:**
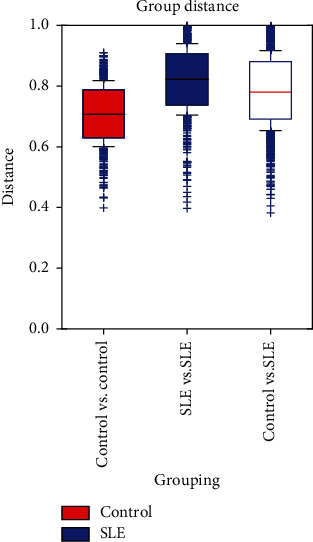
Bray-Curtis statistical algorithm was used to calculate the distance between each sample and obtain the distance matrix. The distance between the SLE group and the HC group with quartiles was calculated, and a box diagram of the distance between the two groups was drawn to compare the distance distribution differences. Student's two-sample *t*-test method was used to compare the distance between each group.

As we can see in Figure 1, the sample distance of the control vs. control was significantly shorter than SLE vs. SLE group and control vs. SLE group (*p* < 0.05). Interestingly, although the alpha diversity of SLE patients remained unchanged compared with that of healthy controls, the sample distance of the former was obviously longer, suggesting healthy children possess microbiota with more similar species composition and the heterogeneity of the microbiota among SLE patients.

**Figure 2:**
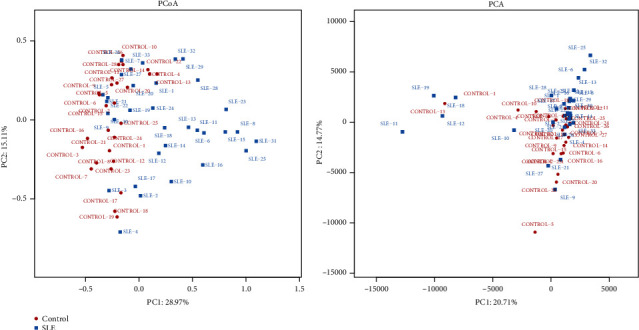
PCoA analysis and PCA analysis were conducted by R version 3.5.1. The red dots represent health controls, and the blue dots represent systemic lupus erythematosus patient samples. PCoA analysis sorts a series of eigenvalues and eigenvectors. It selects the most important eigenvalues ranked in the first few and shows them in the coordinate system. The result can be regarded as a rotation of the distance matrix. The red dots and the blue dots distribute in different clusters on the coordinate axis, revealing a clear separation between the SLE and HC groups. PCA analysis uses variance decomposition to reflect the differences of multiple groups of data on the two-dimensional coordinate graph. The PCA analysis of our study did not show clear separation.

A scatter plot based on principal coordinates analysis (PCoA) revealed a clear separation between the SLE and HC groups (Figure 2). It should be highlighted that a principal component analysis (PCA) of SLE and HC subjects, based on 16 rRNA microbiota profiles, did not show distinct clustering patterns. We witnessed the association between gastrointestinal microbiota and immune factors at the level of bacterial composition but not at the level of the metabolite landscape of gut microbiota, which suggests that SLE can influence the heterogeneous species inhabiting the gut but not the metabolic pattern [20].

Twenty phyla were detected, among which Firmicutes, Bacteroidetes, Proteobacteria, Actinobacteria, Verrucomicrobia, Fusobacteria, and Synergistetes were the most abundant, with Firmicutes being the most dominant. At the genus level, a total of 380 genera were detected in all the samples, and the top 10 genera with the largest abundance were *Faecalibacterium* (average abundance was 17.26%), *Agathobacter* (6.23%), *Roseburia* (5.45%), *Megamonas* (5.37%), *Lachnoclostridium* (3.45%), *Subdoligranululum* (2.58%), *Bacteroides* of Bacteroidetes (7.14%), *Prevotella 9* (5.46%), *Escherichia-Shigella* (Proteobacteria, average abundance was 3.97%), and *Bifidobacterium* (Actinomycetes, average abundance was 2.79%).

Differently abundant species in both groups are listed in Table 2. There were a total of 54 bacteria, including 1 phylum, 3 classes, 5 orders, 8 families, and 37 genera. Proteobacteria was the only different bacterium at the phylum level, increasing in SLE patients. *Gammaproteobacteria*, *Bacilli*, *Enterobacteriaceae*, *Escherichia_Shigella*, *Ruminococcus_gnavus_group*, *Lachnoclostridium*, and *Kluyvera* were also more abundant in SLE patients, while Ruminococcaceae, *Agathobacter*, *Ruminococcus_2*, *Coprococcus_2*, *Eubacterium_coprostanoligenes_group*, *Dialister*, *Faecalibacterium*, and *Subdoligranulum* were distinctly richer among the healthy controls.

**Figure 3:**
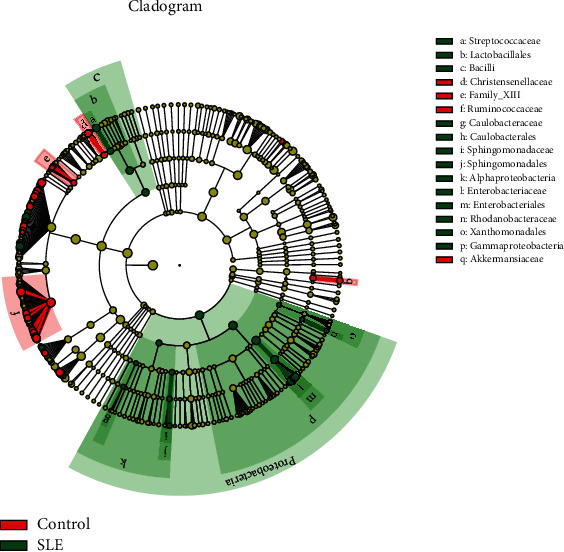
Differential analysis using LEfSe software. Red nodes represent the microbial group that plays an important role in the HC group, green nodes represent the microbial group that plays an important role in the SLE group, and yellow nodes represent the microbial group that does not play an important role in both groups.

Using LEfSe software, a nonparametric factorial Kruskal-Wallis (KW) sum-rank test was conducted to identify species with distinct abundance differences before undertaking linear discriminant analysis (LDA) to estimate the influence of each component (species) on the difference. The difference in microflora from phylum to genus was determined by an LDA value greater than 2 and a *p* value less than 0.05 (Table 2). We can see that for SLE patients, Proteobacteria at the phylum level, Gammaproteobacteria at the class level, Enterobacteriales at the order level, and Enterobacteriaceae at the family level are the most relevant components, and they possess a subordinate relationship. On the other hand, Ruminococcaceae, Christensenellaceae, and Family_XIII at the family level and *Akkermansiaceae* at the genus level were abundant in the healthy controls (Figure 3).

The nonparametric factorial Kruskal-Wallis sum-rank test was used for calculating the difference of abundance. Log value of abundance reflects the abundance of the different species. LDA value estimates the influence of the abundance of each component (species) on the overall abundance differences. *p* < 0.05 and LDA > 2 represent significantly different species.

### 3.3. Different Metabolites' Analysis by GC-MS

Variable importance for the projection (VIP) obtained from the OPLS-DA model was used to measure the influence intensity and explanatory ability of the expression pattern of each metabolite on the classification and discrimination of samples of each group, and the different metabolites with biological significance were excavated.

This study defined metabolites with VIP > 1 in the multidimensional statistical analysis and *p* < 0.05 in univariate statistical analysis as significantly different metabolites, including 24 in positive ion mode and 37 in anion mode. Ruling out 6 overlapping data, there were a total of 55 significantly different metabolites (Table 3), including mainly long-chain fatty acids and amino acids. Among them, cyclohexylsulfamate had the highest VIP value, indicating that its decline had a greater impact on the occurrence of SLE diseases.

**Figure 4:**
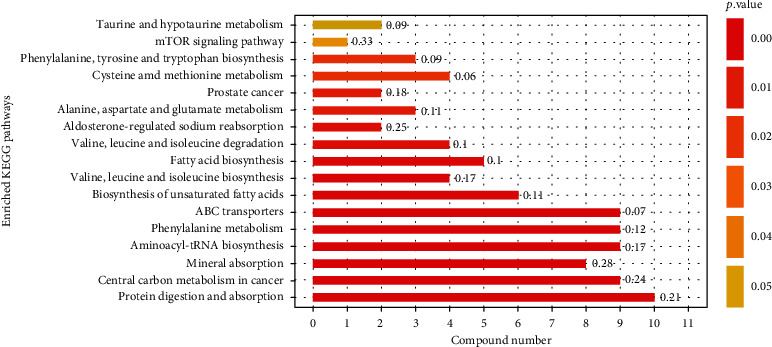
KEGG pathways. Numbers on the right of the bar are rich factor, reflecting the degree of enrichment (rich factor = the number of genes enriched in this pathway/the number of all genes in this pathway).

Of the 55 SLE-altered metabolites submitted to the KEGG (Kyoto Encyclopedia of Genes and Genomes) website (http://www.kegg.jp/), we were able to match 27 of them with 66 pathways. The *p* value represents the significance of enrichment of the KEGG pathway, and the number of differentially expressed metabolites contained in the KEGG pathway reflects the degree of influence of SLE on each pathway to some extent (Figure 4). Taking two factors into consideration, protein digestion and absorption (*p* = 1.75*E* − 9) were the most significant among all these pathways, involving *L*-tryptophan, tyramine, *L*-phenylalanine, *L*-leucine, *L*-methionine, *L*-alanine, *L*-glutamine, *L*-valine, *L*-isoleucine, and *L*-tyrosine.

**Figure 5:**
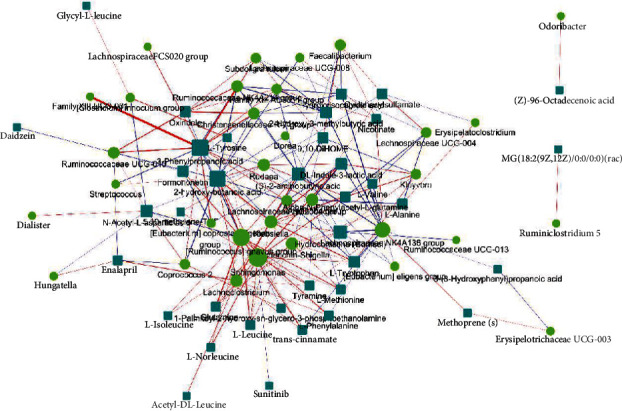
Circles represent significantly different genera, and rectangles represent significantly different metabolites. The color of the line represents the positive and negative value of correlation coefficient (blue represents a negative correlation and red represents a positive correlation), and the thickness of the line is proportional to the absolute value of the correlation coefficient. The node size is positively correlated with its degree (the greater the degree, the larger the node size).

The network of metabolites and intestinal flora using Cytoscape version 3.5.1 is shown in Figure 5. We can see that the most central location belongs to 3-phenylpropanoic acid, which exhibits a strong positive correlation with *Family XIII UCG-001* and a negative correlation with all 5 differentially expressed genera of Proteobacteria. *Sphingomonas* of the Proteobacteria phylum plays the most important role as a bacterium, and it is obviously positively associated with all 10 metabolites in the protein digestion and absorption pathway mentioned above.

## 4. Discussion

The current study found that alpha diversity of the SLE group is not different when compared to healthy controls, and the beta diversity was partially altered, which was in accordance with a previous study among adult SLE patients [20]. The fact that the alpha diversity is not statistically different between the healthy control group and the group affected by the pathology could be related to age-dependent factors and also to the small number of children in the analysis. To be precise, PCoA successfully identified 2 separated clusters of SLE and HC subjects when PCA did not, indicating that the changes induced by SLE became marked at the functional level, i.e., the level of microbial population structure (or 16S rRNA), regardless of the heterogeneities that appeared above at the highest level of the functional hierarchy, i.e., the metabolite level (Figure 1). In contrast to our outcomes on the alpha and beta diversity, the outcomes of a study by Zhu revealed upregulated alpha diversity in the SLE group; in addition, Rojo et al. reported beta diversity in PCA but not PCoA [16, 21]. This inconsistency may be due to age and sex differences since previous papers contained only adult patients and fewer or no male patients.

Another important finding was the increase in Proteobacteria and Enterobacteriales at the phylum and family levels among SLE patients in our project in southern China, and this finding was also reported by Wei et al., He et al., and Hevia and Milani from northern China. However, results from studies conducted in Spain and southern China do not support our results [22–24]. In Spain, the intestinal flora of SLE patients was characterized by a significantly lower Firmicutes/Bacteroidetes ratio and showed a depletion of *Lachnospiraceae* and *Ruminococcaceae* and an enrichment of *Bacteroidaceae* and *Prevotellaceae* [24]. In southern China, the intestinal flora of SLE patients was characterized by a significant increase in Actinobacteria [22]. It can therefore be assumed that the flora composition is irrelevant to the geographical location. On the other hand, consistent with the literature, this research found that Ruminococcaceae levels were higher in healthy controls [21, 23, 24]. Moreover, there was a list of other genus assigned to the Lachnospiraceae family that are widely descripted as beneficial bacteria in literature elevated in health controls, including Lachnospiraceae_UCG_008 and Lachnospiraceae_UCG_004 [25, 26]. Additionally, previous research has established that the increase in Proteobacteria and the decrease in Ruminococcaceae were associated with lupus nephritis with gastrointestinal damage [27]. Na et al. proposed that an increased prevalence of the bacterial phylum Proteobacteria is a marker for an unstable microbial community (dysbiosis) and a potential diagnostic criterion for disease [28]. Shang et al. identified Ruminococcaceae to be responsible for the degradation of diverse polysaccharides and fibres [29].

An obvious finding to emerge from the analysis is that amino acids were enriched in SLE faecal samples compared to HCs (Table 3), which was in accordance with previous research that found that their plasma concentration decreased [30–33]. Using gas chromatography-mass spectrometry, Yan et al. were able to detect the serum depletion of 12 amino acids, including tryptophan, alanine, proline, glycine, serine, threonine, aspartate, glutamine, asparagine, lysine, histidine, and tyrosine [30]. Similarly, Ouyang et al. found that *L*-alanine, *L*-isoleucine, *L*-lysine, *L*-phenylalanine, *L*-tyrosine, and *L*-valine were downregulated in the plasma of SLE patients [31]. Notably, four essential amino acids, *L*-valine, *L*-leucine, *L*-phenylalanine, and *L*-tryptophan, were listed among the metabolites depleted in plasma but enriched in faeces. These four amino acids have important physiological functions, such as the regulation of immunity, metabolism, and neural activity. Choi et al. reported that low dietary tryptophan prevented autoimmune pathology in lupus-prone mice, whereas high dietary tryptophan exacerbated disease [32]. Moreover, glucogenic amino acids, such as proline and *L*-methionine, and glucogenic and ketogenic amino acids, such as *L*-tyrosine, were increased in the faeces of SLE patients, indicating that there might be disorders in glucose metabolism and energy metabolism, as these amino acids could emerge as potential energy sources [33, 34]. Regarding the fatty acids, we found the abundance varied, as most long-chain fatty acids decreased while short-chain fatty acids increased in SLE children. This finding is contrary to a recent study that reported enriched fatty acids in the faeces of the SLE group [35]. Another study also established that long-chain fatty acids were less abundant in SLE patient serum [36]. At least half of SLE patients have gastrointestinal symptoms, including nausea, vomiting, anorexia, abdominal pain, diarrhoea, and abdominal distension, which may lead to an imbalance of amino acids and fatty acids [37].

The results of this study show that SLE-enriched faecal amino acids were significantly located in the protein digestion and absorption, ABC transporters, and aminoacyl-tRNA biosynthesis pathways. Amino acids are mainly absorbed in human intestinal mucosal cells through carrier proteins and the *γ*-glutamine cycle. Through type I ABC transporters in the third subgroup, intestinal prokaryotes and archaea obtain amino acids, which can be used for the synthesis of microbial proteins, including the biosynthesis of aminoacyl-tRNA, microbial energy metabolism, and conversion into a variety of physiologically active substances, such as neurotransmitters and hormones [38]. As a result, the increase in amino acids in faeces can impact the physiological activities of microorganisms and their hosts. For example, SLE-rich faecal branched-chain amino acids, including *L*-leucine and *L*-valine, can be used as precursors of membrane fatty acids, as well as key synergistic regulators of pathogen growth and virulence. Low-proline or low-protein diets in germ-free mice parasitized by human intestinal flora led to reduced expansion of wild-type *C. difficile* after challenge, indicating that the effectiveness of amino acids may be important for *C. difficile* infection [39]. Tryptophan is essential for the growth of certain pathogens, and severe tryptophan deficiency may hinder normal *Chlamydia trachomatis* onset and reduce its activation [40].

Of association analysis, this study found that *Sphingomonas* plays the most important role in the network of metabolites and intestinal flora. We found higher levels of the bacterium in SLE children. It is encouraging to compare this rise with that found by Corrêa et al., who found that *Sphingomonas* was at lower relative levels in healthy periodontal sites of SLE patients [41]. Chen et al.'s research provided evidence for increased microbial transmission along the gastrointestinal tract of most certain species in SLE patients compared to HCs, implicating a damaged barrier in the upper gastrointestinal tract in SLE patients who possibly cause transmissible oral species to colonize the gut [42].

It is unfortunate that most studies concerning gut microbiota and metabolites in SLE and LN patients include a study cohort of only adults, so our literature search may not be comprehensive enough. There is also a potential confounding effect of medications, as most patients were receiving treatment at the time of sample collection, and a large list of medications is postulated to affect the balance of taxa within the gut microbiome community [43, 44]. Another limitation of this study is that the SLE group and the HC group were not perfectly matched.

## 5. Conclusion

This study set out to investigate intestinal flora and metabolomics in SLE children. This study has shown that Proteobacteria was increased in SLE patients. Of this phylum, the genus *Sphingomonas* plays a very important role, as it exerts a significant positive correlation with a series of proteins in the protein digestion and absorption pathway. *Sphingomonas* were reported to be less abundant in healthy periodontal sites of SLE patients than in those of HCs, indicating the transmission of oral species to the gut. Adjunct therapeutic modalities of adjusting microbiota can be innocuous and worth trying. Considerably, more work will need to be done to determine the pathogenesis and treatment of SLE in children.

## Figures and Tables

**Table 1:** Characteristics of the study cohorts.

	SLE	HC
Fecal samples	33	28
Female	26 (78.79%)	14 (50%)
Age, years, mean ± SD	12.39 ± 2.40	10.61 ± 3.67
BMI, kg/m^2^, mean ± SD	18.79 ± 2.30	18.57 ± 4.51
DISEASE ACTIVITY PARAMETERS IN SERUM		
Elevated ESR, mm/h	11 (33.33%)	
Elevated CRP, mg/L	1 (0.30%)	
Reduced complement C3	18 (54.55%)	
Reduced complement C4	17 (51.52%)	
Positive ANA	21 (63.64%)	
Positive ANCA	2 (6.06%)	
Positive C1q	16 (48.48%)	
SLEDAI SCORE		
No or mild activity (0-4)	22 (66.67%)	
Moderate activity (5-9)	2 (6.06%)	
High activity (10-14)	9 (27.27%)	
Very high activity (≥15)	0	
TREATMENT		
GCs + HCQ	3 (9.09%)	
GCs + HCQ + CTX	12 (36.36%)	
GCs + HCQ + CsA + CTX	2 (6.06%)	
GCs + HCQ + MMF	15 (45.45%)	
GCs + HCQ + CTX + MMF	1 (3.03%)	

SD: standard deviation, ESR: erythrocyte sedimentation rate, CRP: C-reactive protein, ANA: antinuclear antibody, ANCA: antineutrophil cytoplasmic antibody, C1q: anti-C1q antibody. GCs: glucocorticoids, HCQ: hydroxychloroquine, CTX: cyclophosphamide, CsA: cyclosporin A, MMF: mycophenolate mofetil.

**Table 2:** Different species.

Flora category	Log value of abundance	Group	LDA value	*p* value	Level
Proteobacteria	4.967	SLE	4.49	0.007	Phylum
Alphaproteobacteria	2.883	SLE	3.255	0	Class
Gammaproteobacteria	4.95061	SLE	4.486	0.007	Class
Bacilli	4.154298	SLE	3.656	0.006	Class
Enterobacteriales	4.92	SLE	4.495	0.006	Order
Xanthomonadales	2.178	SLE	3.563	0	Order
Caulobacterales	1.81	SLE	3.61	0.001	Order
Sphingomonadales	2.497641	SLE	3.013	0	Order
Lactobacillales	4.152046	SLE	3.655	0.008	Order
Ruminococcaceae	5.556	HC	4.821	0.005	Family
Streptococcaceae	3.838	SLE	3.284	0.017	Family
Christensenellaceae	3.909	HC	3.377	0.04	Family
Caulobacteraceae	1.788	SLE	3.589	0.001	Family
Enterobacteriaceae	4.920289	SLE	4.495	0.006	Family
Family_XIII	2.853446	HC	2.797	0.036	Family
Sphingomonadaceae	2.497641	SLE	3.021	0	Family
Rhodanobacteraceae	1.816683	SLE	3.679	0	Family
Erysipelotrichaceae_UCG_003	3.358	HC	2.768	0.003	Genus
Lachnospiraceae_UCG_008	1.772055	HC	3.586	0.043	Genus
Lachnospiraceae_UCG_004	3.600504	HC	2.964	0.025	Genus
Ruminococcaceae_NK4A214_group	3.618	HC	3.006	0.006	Genus
Lachnospiraceae_FCS020_group	2.815	HC	2.738	0.033	Genus
Family_XIII.Family_XIII_UCG_001	2.302	HC	3.209	0.01	Genus
Ruminococcaceae_UCG_013	3.423	HC	2.885	0.002	Genus
Ruminococcaceae_UCG_010	3.021	HC	2.775	0.03	Genus
Lachnospiraceae_NC2004_group	2.246211	HC	3.241	0.034	Genus
Christensenellaceae_R_7_group	3.89737	HC	3.376	0.021	Genus
Family_XIII_AD3011_group	2.580169	HC	3.234	0.037	Genus
Lachnospiraceae_NK4A136_group	3.869956	HC	3.367	0.001	Genus
Escherichia_Shigella	4.809	SLE	4.393	0.019	Genus
Agathobacter	4.932	HC	4.313	0.004	Genus
Akkermansiaceae	3.328	HC	2.979	0.007	Genus
Ruminococcus_2	4.314	HC	3.767	0.049	Genus
Clostridium_innocuum_group	2.778	SLE	2.938	0.037	Genus
Fusicatenibacter	3.892	HC	3.342	0.019	Genus
Streptococcus	3.829	SLE	3.271	0.032	Genus
Dorea	3.792	HC	3.122	0	Genus
Coprococcus_2	4.047	HC	3.686	0	Genus
Hungatella	3.387	SLE	3.087	0.033	Genus
Erysipelatoclostridium	3.226	SLE	2.98	0.017	Genus
Eubacterium_coprostanoligenes_group	4.435	HC	3.809	0.008	Genus
Ruminococcus_gnavus_group	4.308	SLE	3.958	0.003	Genus
Dialister	4.603	HC	4.101	0.001	Genus
Klebsiella	3.669467	SLE	3.274	0	Genus
Lachnoclostridium	4.698468	SLE	4.244	0.027	Genus
Faecalibacterium	5.329871	HC	4.598	0.015	Genus
Ruminiclostridium_5	3.464955	HC	2.995	0.014	Genus
Rudaea	1.816683	SLE	3.68	0	Genus
Sphingomonas	2.425476	SLE	3.099	0	Genus
Kluyvera	4.006708	SLE	3.48	0.001	Genus
Subdoligranulum	4.57793	HC	4.024	0.012	Genus
Odoribacter	2.434041	HC	3.256	0.048	Genus
Akkermansia	3.327618	HC	2.973	0.007	Genus
Eubacterium_eligens_group	3.929	HC	3.478	0.022	Genus

SLE: systemic lupus erythematosus group; HC: health control group; LDA: linear discriminant analysis.

**Table 3:** Significantly different metabolites.

Description	VIP	Fold change (SLE/HC)	*p* value	*m*/*z*	rt (s)
(S)-2-Aminobutyric acid	1.134457	7.300207	0.027901	102.0559	714.909
(Z)-6-Octadecenoic acid	1.460343	0.604532	0.00674	282.2502	91.72
16-Hydroxypalmitic acid	1.874055	0.499351	0.009079	314.2681	92.142
1-Oleoyl-sn-glycero-3-phosphocholine	1.904087	0.331859	0.032946	522.3537	354.483
1-Palmitoyl-2-hydroxy-sn-glycero-3-phosphoethanolamine	2.796585	0.225656	7.95*E* − 06	454.2913	372.123
2-Hydroxy-3-methylbutyric acid	4.457651	4.059644	0.024633	117.0556	283.2115
2-Hydroxy-butanoic acid	1.365271	2.252586	0.000967	103.0398	364.888
3-(3-Hydroxyphenyl)propanoic acid	7.504876	3.292347	0.042029	165.0556	213.446
3-Phenylpropanoic acid	8.186158	0.482206	0.018599	149.0607	197.716
5,10-Methylene-THF	2.02514	5.177854	0.038319	456.1695	81.174
9,10-DiHOME	1.759624	0.622241	0.018751	313.2384	197.908
Acetyl-DL-leucine	2.436625	3.258067	0.020322	172.0978	372.417
all *cis*-(6,9,12)-Linolenic acid	1.996995	0.552529	0.003271	279.2309	91.4225
Alpha-linolenic acid	1.125754	0.495219	0.027657	296.2578	85.836
Alpha-N-phenylacetyl-L-glutamine	1.032981	6.993584	0.03739	263.1035	434.981
Bisindolylmaleimide I	1.251916	0.533825	0.034091	411.1837	60.485
*cis*-9-Palmitoleic acid	2.112812	0.530253	0.010487	315.2523	157.6505
Cyclohexylsulfamate	18.63932	0.399938	0.01068	178.0541	134.362
Daidzein	1.860198	0.37232	0.049911	253.0506	90.079
DL-Indole-3-lactic acid	1.389089	1.868281	2.75*E* − 05	188.0698	482.9055
DL-Methionine sulfoxide	1.188165	1.570971	0.013233	164.0387	705.022
Enalapril	2.770035	3.292851	0.02267	375.1845	335.5975
Formononetin	1.159497	12.16737	0.031198	269.08	75.902
Formylanthranilic acid	1.108129	1.780982	0.030492	164.035	124.961
Gamma-L-glutamyl-L-valine	1.260303	1.995892	0.001099	245.1144	738.78
Glycyl-L-leucine	1.714816	1.468498	0.009308	187.1089	549.388
Hydrocortisone (cortisol)	2.126813	6.47518	0.003054	421.2224	94.857
Hydroxyisocaproic acid	10.89455	4.366298	0.014686	131.0711	244.439
*L*-Alanine	1.917025	1.859043	0.000362	88.04036	663.2525
*L*-Glutamine	1.81976	2.137778	0.00051	145.0619	715.39
Linoleic acid	3.644522	0.377719	4.77*E* − 06	298.2736	84.196
*L*-Isoleucine	1.163985	4.459251	0.006748	261.182	497.738
*L*-Leucine	7.61484	1.835031	9.53*E* − 05	130.0874	497.981
*L*-Methionine	2.024436	2.05734	0.005488	148.0437	536.5665
*L*-Norleucine	1.193737	5.606042	0.047861	263.1959	493.5235
*L*-Phenylalanine	6.291687	1.785839	0.000231	164.072	482.6735
*L*-Tryptophan	2.780279	1.785305	2.45*E* − 05	203.0828	482.5715
*L*-Tyrosine	3.001494	1.658265	0.006866	180.0666	569.619
*L*-Valine	3.438313	3.006204	0.000931	116.0716	574.471
Methoprene (S)	1.236084	0.334761	0.004683	328.2844	64.9405
MG(18 : 2(9Z,12Z)/0 : 0/0 : 0)[rac]	3.617466	0.313071	7.55*E* − 05	355.2834	69.542
N-Acetyl-L-aspartic acid	1.020191	0.48076	0.016099	174.0408	773.394
N-Acetylneuraminic acid	1.083761	0.539489	0.027489	310.1126	716.366
Nicotinate	1.873086	0.543751	0.001525	124.0383	412.64
Oleic acid	1.514581	0.359758	0.001028	265.2517	120.4355
Oxindole	1.01039	0.407327	0.033063	134.0594	80.262
Palmitic acid	4.166731	0.485449	0.035091	255.2329	88.202
p-Hydroxyphenylacetic acid	1.625958	2.672582	0.032862	151.0399	386.525
Pregnenolone sulfate	1.984757	0.31761	0.008648	395.1888	55.708
Stearidonic acid	2.760427	23.20345	0.040155	337.2366	372.7625
Sunitinib	3.289336	0.283469	0.002701	397.2046	61.461
*trans*-Cinnamate	1.407338	1.766262	0.000204	147.045	482.794
*trans*-Vaccenic acid	3.069738	0.496621	0.012385	283.2626	93.176
Tridecanoic acid (tridecylic acid)	1.351245	0.338885	0.032456	213.186	91.512
Tyramine	2.011381	1.73279	0.001232	120.0798	479.944
Vanillin	1.308743	1.345909	0.016492	151.0398	75.058

VIP: variable importance for the projection; *m*/*z*: mass-to-charge ratio; rt (s): retention time. The VIP value was calculated based on orthogonal partial least squares discriminant analysis to measure the influence and explanatory ability of each metabolite expression pattern on the classification discrimination of each group of samples, and to excavate the different metabolites with biological significance. Fold change describes the ratio of metabolite content (SLE/HC). *p* value < 0.05 represents that metabolites have significant difference in one-way ANOVA or two-way ANOVA analysis.

## Data Availability

All data are available in our manuscript.
